# Structural Bicortical Autologous Iliac Crest Bone Graft Combined with the Tunnel Bone Tamping Method for the Depressed Tibial Plateau Fractures

**DOI:** 10.1155/2021/1249734

**Published:** 2021-08-24

**Authors:** Zhongzheng Wang, Yanbin Zhu, Xiangtian Deng, Xin Xing, Siyu Tian, Lei Fu, Xiaoli Yan, Wei Chen, Zhiyong Hou, Yingze Zhang

**Affiliations:** ^1^Department of Orthopaedic Surgery, Third Hospital of Hebei Medical University, Shijiazhuang, Hebei, China 050051; ^2^Key Laboratory of Biomechanics of Hebei Province, Shijiazhuang, Hebei, China 050051; ^3^NHC Key Laboratory of Intelligent Orthopaedic Equipment, Shijiazhuang, Hebei, China 050051; ^4^School of Medicine, Nankai University, Tianjin, China 300071

## Abstract

**Background:**

Clinically, autologous iliac crest bone grafts (ICBG) and bone tamping methods are often applied to manage depressed tibial plateau fractures (DTPFs). The purpose of this study was to describe and evaluate the technique of using structural bicortical autologous ICBG combined with the tunnel bone tamping method (TBTM) for treating DTPFs.

**Methods:**

All patients with DTPFs who underwent structural bicortical autologous ICBG combined with TBTM from January 2016 to February 2018 were prospectively analysed. Demographics, injury, surgery, postoperative complications, and clinical outcomes were recorded. All patients were followed up for more than 30 months. Postoperative radiography and CT were employed to assess fracture healing and the reduction quality.

**Results:**

Forty-three of the included patients completed the follow-up. No malreduction was observed. Based on the immediate postoperative imaging, the intra-articular step-off was significantly reduced (8.19 mm preoperatively vs. 1.30 mm immediate postoperatively, *P* < 0.001). From the immediate operation to the latest follow-up, the reduction was maintained significantly well, with a nonnegligible absolute difference (0.18 mm, *P* = 0.108). A remarkable secondary loss of reduction (intra-articular step off > 3 mm) was found in two elderly patients (2/43, 4.65%). The incidence of complications related to the bone-graft donor and bone-graft site was 2.33% and 4.65%, respectively. At the final follow-up, the mean Hospital for Special Surgery (HSS) score of the knee was 98.19 ± 2.89, and the mean 36-Item Short-Form Health Survey (SF-36) score was 95.65 ± 4.59.

**Conclusion:**

Structural bicortical autologous ICBG combined with TBTM is radiologically effective and stable in terms of complications for the DTPFs.

## 1. Introduction

Depressed tibial plateau fractures (DTPFs) are the most common type of tibial plateau fractures (TPFs), typically involving type II–VI fractures according to the Schatzker classification system [1–3]. Surgical management of DTPFs involves accurate reconstruction of the articular surface and stable fixation to prevent the development of secondary knee osteoarthritis [4, 5]. Currently, the gold standard method is a retrograde elevation of the depressed articular fragments, filling in the residual defect zone with autologous cancellous bone, allogeneic bone, or synthetic bone material, and subsequently plating it for stabilization [6–8].

Structural autologous iliac crest bone grafts (ICBGs) are the most popular bone grafts because they provide mechanical strength for fixation stabilization and strong potential for osteogenesis [9], despite a higher incidence of complications [10, 11]. In contrast, nonstructural autologous cancellous bone grafts or corticocancellous bone grafts of the iliac crest cannot provide adequate mechanical stability to allow for early knee movement, regardless of the fixation method [8, 12, 13]. In our practice, we prefer to use structural bicortical autologous ICBG, which in theory can not only provide mechanical support as does cortical bone but also substantially contribute to bony healing due to the embedded large amount of cancellous bone and red bone marrow. In fact, most complications encountered can be effectively avoided if one has adequate familiarity with the anatomical and physiologic characteristics of the iliac crest and procedural refinements [9, 11, 14, 15].

To the best of our knowledge, although the tunnel bone tamping method (TBTM) has been widely used in practice for reducing the depressed articular fragments of TPFs, its role in the quality of reduction and maintaining the reduction until bony union remains controversial, and the clinical outcomes have not been clarified to date [7, 8, 16]. In this study, we aimed first to evaluate the role of structural bicortical autologous ICBG combined with TBTM in achieving and maintaining reduction until bony union for the treatment of DTPFs, and second, to investigate the clinical outcomes.

## 2. Methods

### 2.1. Study Design and Participants

From January 2016 to February 2018, consecutive patients with DTPFs were recruited at our level-I trauma centre for this study. DTPF was defined as a depression of articular cartilage fragments of the tibial plateau greater than 2 mm. The inclusion criteria were as follows: (1) adult patients (age ≥ 18 years); (2) diagnosis of DTPFs; (3) agreement to receive structural bicortical autologous ICBG combined with TBTM treatment and subsequent internal fixation; and (4) agreement to participate in regular follow-up after surgery. The exclusion criteria were as follows: (1) open or pathologic fractures; (2) bone metabolic disease, previous ICBG, infection, or soft tissue injury of the iliac bone donor site; (3) pelvic fractures or bone tumours, associated peripheral nerve injury; or (4) follow-up time less than 30 months.

This study was registered at (NCT04807062) and approved by the Ethics Committee of the participating institution (Theoretical No. 2015-003-1). In accordance with the Helsinki Declaration, informed consent was obtained from all participants. This work has been reported according to the PROCESS criteria [17].

### 2.2. Preoperative Preparation

After admission, the patient's systemic condition, knee swelling degree, and soft tissue injury were evaluated. Relevant examinations, including radiographs, CT scans and MRI scans of the knee, and laboratory haematology tests, were performed to confirm the diagnosis. Then, the therapeutic regimen was selected and a detailed preoperative plan was developed.

**Figure 1:**
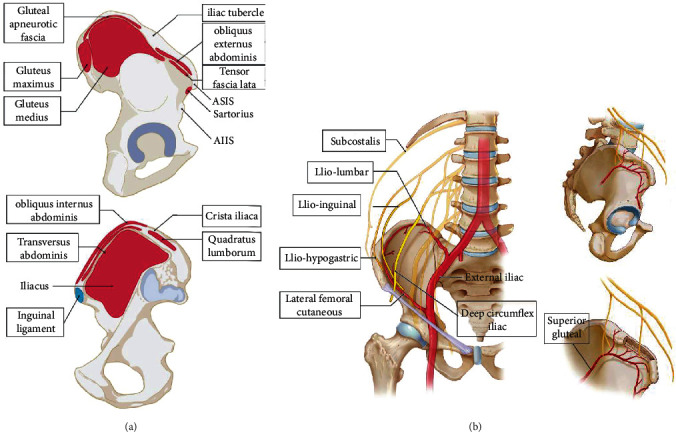
Important anatomical structures of the iliac crest. Iliac crest is located on the upper edge of ilium, which is an arcuate irregular bone structure. The important anatomical structures around the iliac crest can be divided as: (a) surface bony markers and muscle origins and (b) nerve and artery origins.

The iliac crest is the most common donor site for bone grafts and is a rich source of cortical and cancellous bone. Fully understanding the anatomical characteristics of the iliac crest is necessary. The anterior superior iliac spine (ASIS), anterior inferior iliac spine (AIIS), iliac tubercle, and posterior superior iliac spine (PSIS) are important surface markers for evaluating the position of the iliac crest [18, 19]. Many soft tissues, such as the sartorius, tensor fascia lata, superficial layer iliaotibial band, gluteal aponeurotic fascia, inter- and external oblique muscles, transversus abdominis, and iliac muscle, are attached to the iliac crest [19, 20]. In addition, there are many major nerves and arteries around the iliac crest, including the lateral femoral cutaneous nerve, ilio-inguinal nerve, ilio-hypogastric nerve, subcostalis nerve, ilio-lumbar artery, deep circumflex iliac artery, fourth lumbar artery, and superior gluteal artery [21–23]. The detailed anatomy is shown in Figure 1.

### 2.3. Surgical Technique

The surgical procedures were performed by the same team, and all surgeons had more than 10 years of experience in orthopaedic trauma surgery. Before surgery, general or spinal anaesthesia and routine antibiotic prophylaxis were administered. The supine position and raising the hip with a thick pad were applied during surgery.

**Figure 2:**
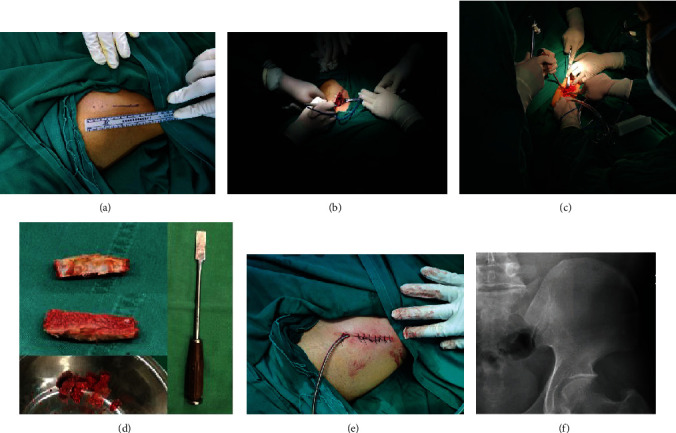
Steps to obtain the structural bicortical autologous iliac crest bone. (a) Locate the bony markers of the iliac crest and predict the surgical incision; (b) soft tissues were dissected along the lateral lip of the iliac crest with an electrome; (c) a special sharp narrow chisel was used to harvest a bicortical bone segment; (d) structural bicortical autologous iliac crest bone segment, cancellous bone, and bone harvest tool; (e) surgical incision at the bone-graft donor site; (f) postoperative radiograph of the bone-graft donor site.

First, a 4 cm long oblique skin incision was made, starting 3 cm behind the ASIS and extending back along the iliac crest. The gap between the gluteal aponeurosis and external oblique muscle was revealed. An electrome was used to dissect the gluteal aponeurosis downward along the lateral lip of the iliac crest approximately 1 cm and to dissect the external oblique muscle inward approximately 1 cm. The tissue planes were preserved without revealing the inner table of the iliac bone and iliac muscle. A special sharp narrow chisel was used to harvest bicortical bone segments containing the lateral table of the ilium and part of the iliac crest. In addition, the cancellous bone at the bottom can be harvested as needed. After bone harvesting, an absorbable haemostatic gelatine sponge (Xiangen®, China) was used to fill the donor site. Before incision closure, the soft tissue planes were sutured back for reconstruction, and a drainage was placed (Figure 2).

**Figure 3:**
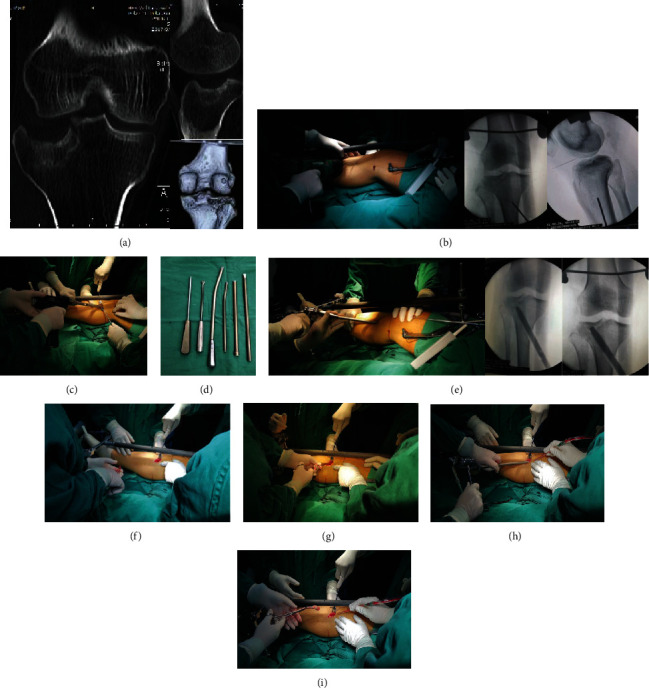
Steps of the reduction of the depressed articular fragments via TBTM and filling of the structural bicortical autologous iliac crest bone segment. (a) Knee CT scans of a 42-year-old man with DTPF; (b) a syringe needle was used to locate the articular cavity of the knee, and a 2.5 mm Kirschner wire was used to locate the position of the depressed area; (c) a power-driven trephine was used to create a bone tunnel on the anterior proximal tibia; (d) cylindrical metal tamps of different shapes and sizes; (e) retrograde reduction of the depressed articular fragments; (f) after reduction, cancellous bone was filled beneath the depressed articular fragments; (g)–(i) the structural bicortical autologous iliac crest bone segment was placed and compressed with a metal bone tamper. Then, reconstruction of the tibial cortical bone window was performed.

Second, a universal external fixator or retractor and a proximal tourniquet of the lower extremity were used for the affected limb. Typically, a syringe needle was used to locate the articular cavity of the knee, and a 2.5 mm Kirschner wire was used to position the depressed area. Then, a cortical window was created using a power-driven hollow trephine on the anterior proximal tibia, and a bony tunnel was established to insert a large metal tamp. Typically, we prefer to create a cortical window on the anteromedial surface of the tibia, no matter what type of fracture, because the anteromedial muscles are less attached. If there is local damage to the anteromedial skin of the tibia, it is also possible to create a cortical window on the anterolateral side. We call the retained round cortical bone block in the middle of the hollow trephine as a cortical bone cap, which was stored for later use. The depressed articular fragments were reduced by applying a series of retrograde impacts with a hammer and different diameters of cylindrical metal tamps, pushing them upward gradually [4, 7, 8]. After reduction, the remaining bone tunnel was filled with cancellous bone and structural bicortical autologous iliac crest bone segment. After the bone strip was pressed into the suitable place with the metal tamp, a bone cap was placed to reconstruct the bone cortex window (Figure 3).

**Figure 4:**
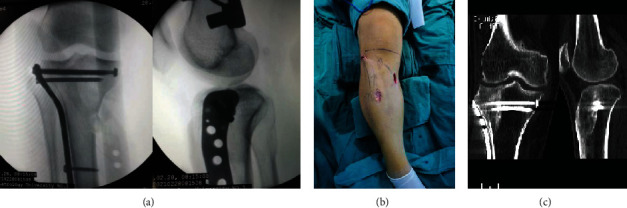
Lateral plate and screw fixation (Schatzker type II) was performed using the minimally invasive percutaneous plate osteosynthesis (MIPPO) technique. (a) Intraoperative fluoroscopy was performed after fixation; (b) surgical incisions; (c) CT scans of the knee during postoperative follow-up.

Third, medial or lateral plate fixation was performed using the minimally invasive percutaneous plate osteosynthesis (MIPPO) technique depending on the position of the depressed articular fragments (typically, Schatzker type II or III—lateral locking plate fixation, and Schatzker type IV—medial locking plate fixation) [24]. Intraoperative C-arm fluoroscopy was performed again to confirm the reduction, complete haemostasis was performed, and the wound was sutured layer by layer (Figure 4).

### 2.4. Postoperative Management and Observational Indexes

Routine antibiotics were used to prevent wound infection within 24 hours postoperatively, and routine subcutaneous injection of low-molecular-weight heparin calcium was used to prevent deep vein thrombosis (DVT) of the lower extremities. The patients immediately underwent postoperative X-ray and CT scans to evaluate the reduction. When the pain subsided, the patients were encouraged to perform nonweight bearing joint movement. At 2 weeks postoperatively, the patients were encouraged to walk with partial weight bearing and slowly proceed to full weight bearing (at 6-8 weeks postoperatively). In addition, imaging examinations, including X-ray (lateral and AP views) of the pelvis and knee and clinical outcome evaluations, were performed at 1, 2, 3, 6, and 12 months after surgery and every 6 months thereafter and obtained the data of the knee CT scans at the final follow-up.

The main outcome measurements included the postoperative malreduction rate, the secondary reduction loss rate, and the bone union time. Two experienced surgeons assessed the quality of the fracture reduction with Picture Archiving and Communication Systems (PACS) software based on the X-ray films and CT scans taken immediately after surgery and final follow-up. A malreduction was defined as an intra-articular step-off of 2 mm or more immediately after the surgery [25]. Secondary reduction loss was defined as an intra-articular step-off greater than 3 mm at the final follow-up compared to the immediate postoperative radiographs and CT scans [26]. At least three cortical unions on radiology were defined as fracture unions [27].

The secondary outcome measurements included operation record information, the incidence of complications at the bone-graft donor and bone-graft sites, and the clinical outcomes. The pain of the bone-graft donors and bone-graft sites was assessed by the visual analog scale (VAS) score (0 representing no pain and 10 representing maximal imaginable pain), the functional outcome of the knee was evaluated by the Hospital for Special Surgery (HSS) score, and patients' overall health status was assessed by the 36-Item Short-Form Health Survey (SF-36) score [22, 28].

### 2.5. Statistical Analysis

Statistical analysis was performed using SPSS version 22.0 (SPSS Inc., Chicago, IL). Continuous variables are presented as the mean values and standard deviation (SD), and categorical variables are expressed as numbers and percentages (%). Univariate analyses were performed using paired *t*-tests applied to differences before and after treatment. A *P* value < 0.05 was considered to be significant.

## 3. Results

### 3.1. Patient Characteristics

In this study, 43 patients out of the 47 selected patients completed the follow-up and were enrolled, including 30 men and 13 women aged between 21 and 67 years (mean age: 46.28 ± 11.82 years). Of them, 26 patients (60.47%) had high-energy injuries, and 28, 8, and 7 had Schatzker II, Schatzker III, and Schatzker IV fractures, respectively. The mean follow-up time was 40.19 ± 7.58 months. The patients' demographics, mechanism of injury, and fracture patterns are shown in Table 1.

### 3.2. Main Outcome Measurements

Table 2 shows that the combined surgical procedure has obvious advantages for DTPFs in terms of postoperative immediate reduction and postoperative long-term maintenance of the reduction in the present study. The results of the paired *t*-test showed that the improvements in intra-articular step-off between preoperation and immediate postoperation were significant (8.19 mm vs. 1.30 mm, *P* < 0.001). From the immediate postoperation to the latest follow-up, the reduction was maintained significantly well, with an unneglectable absolute difference (0.18 mm, *P* = 0.108). In addition, the iliac bone-graft donor and bone-graft sites achieved good bone union in all patients, and the average bone union times were 1.60 ± 0.62 months and 2.45 ± 0.96 months, respectively. No patients had immediate malreduction after surgery. However, a reduction loss occurred in two elderly patients (4.65%) at the final follow-up, but their knee function was not significantly affected (Table 3).

### 3.3. The Secondary Outcome Measurements

Table 3 shows that the combined surgical procedure has obvious advantages for DTPFs in terms of the incision length, operative time, intraoperative blood loss, and time of hospital stay. In our study, one patient suffered continuing pain at the iliac bone-graft donor site for more than six months. After two months of combined treatment with oral NSAIDs and physiotherapy, the pain was significantly relieved. Two patients suffered secondary reduction loss at the bone graft site. Therefore, the incidences of complications of the iliac bone-graft donor and bone-graft sites were 2.33% (1/43) and 4.65% (2/43), respectively. At the final follow-up, the VAS scores of the iliac bone-graft donor and bone-graft sites were 0.21 ± 0.56 and 0.09 ± 0.29, respectively. The mean HSS score of the knee was 98.19 ± 2.98, and the mean SF-36 score was 95.65 ± 4.59.

## 4. Discussion

The results of this study showed that the TBTM could effectively reduce the depressed articular surface of TPFs and that structural bicortical autologous ICBG can provide long-term stability for depressed bone fragments. Lower limb traction and the application of special metal bone tampers were essential to achieve a good reduction. Structural bicortical autologous iliac crest bone can withstand high loads, especially in cases where immediate postoperative stability is prioritized. In addition, the incidence of complications at the bone harvest and bone-graft sites may be reduced with the application of this surgical technique. This research may enrich the understanding of ICBG and TBTM for clinical orthopaedic surgeons.

**Figure 5:**
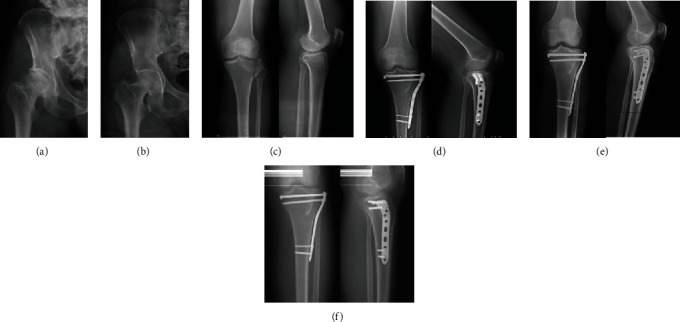
Follow-up of a male patient (43 years old) with DTPF (Schatzker type II). (a) Immediate postoperative radiograph of the bone-graft donor site; (b) radiograph of the bone-graft donor site 1 month after surgery showing the iliac bone healed; (c) preoperative radiographs of the affected knee; (d) immediate postoperative radiographs of the affected knee; (e) postoperative radiographs of the knee 1 month after surgery; (f) postoperative radiographs of the knee 2 month after surgery showing the iliac bone healed.

Although allogeneic bone and synthetic bone materials have been used for treating of TPFs, autologous ICBG remains the standard for clinical bone grafting [9, 11, 13]. Autologous cancellous bone of the iliac crest has strong potential for osteoinduction, osteoacusis, and osteogenesis, retains an intact trabecular structure, and is rich in growth factors. After grafting, it can rapidly incorporate into the host site and accelerate bone formation [29]. Similar to cancellous bone, autologous cortical bone of the iliac crest provides a structurally sound osteoconductive medium with greater mechanical stability than cancellous bone [30]. In addition, the structural bicortical autologous iliac crest bone segment had a uniform yield and a good thickness and volume of cancellous tissue. It can be cut and shaped into the desired configuration, adjusting to the needs of high and sustained load-bearing at the bone-graft site [31]. In clinical practice, the author proved that structural bicortical autologous iliac crest bone intuitively offers the advantages of both immediate mechanical stability from the cortical bone and the osteoinduction and osteogenesis of cancellous bone. The knees of all patients achieved fast bone union (2.45 months) and high HSS scores (98.19 points). The follow-up radiographs of bone-graft donor site and bone-graft site were shown in Figure 5.

The precise reconstruction of the articular surface of the TPFs and the prevention of reduction loss are of decisive importance in the treatment of DTPFs [30]. At present, the standard surgical procedure for DTPFs is to apply a bone tamping technique combined with autologous cancellous bone filling and then fix it with screws or plates [4, 16]. However, the reduction quality and mechanical support effect of this traditional method in the treatment of DTPFs are the main questions of concern [8, 32, 33]. A study by Heiney et al. [6] showed that the incidence of overreduction and malreduction of conventional metal bone tamps was as high as 19.2% and 47.1%, respectively. However, none of the 43 patients with DTPFs we treated by structural bicortical autologous ICBG combined with TBTM had a malreduction, and only 2 (4.65%) suffered a secondary loss of reduction due to severe osteoporosis. The reason for these good results may be that the application of TBTM with lower limb traction would not result in overreduction of the depressed articular fragments, and the structural bicortical autologous ICBG can provide direct postoperative strength and stability to the surrounding tissues to act as a scaffold for subsequent bone growth. In addition, the paired *t*-test results confirmed that our method significantly reduced the intra-articular step-off immediately postoperation compared with preoperation (8.19 mm vs. 1.30 mm, *P* < 0.001) and provided long-term reset stability for the reduced articular fragments (1.30 mm vs. 1.48 mm, *P* = 0.108).

The high complication rate of the iliac bone graft donor site remains a matter of concern to orthopaedic surgeons [11, 34]. Common complications include pain of the bone-graft donor site, sensory disturbances, haematoma, infection, and iliac wing fractures [11]. A large retrospective study by Arrington et al. [35] showed that the incidence of postoperative bone-graft donor site complications was 15.8%. Goulet et al. [36] showed that 33 (38%) of 87 patients suffered pain at 6 months after autogenous iliac bone harvesting, and 11 (13%) patients had ambulatory deficits. Singh et al. [37] retrospectively analysed 46 patients with anterior ICBG with an average follow-up of 41 months. On the VAS score, the average persistent pain at the donor site was 1.7/10. The occurrence of these complications is usually related to injury to adjacent tissues and structures during harvest and to the technique used to the surgical procedure [35]. In fact, many surgeons believe that the incidence of these complications has been overestimated [13–15]. They can be effectively reduced by careful dissection and improving the surgical technique [15]. In a prospective study by Russell and Leighton [13], pain at the iliac bone donor site had resolved within 6-12 months in 38 patients who received autogenous ICBG, and no patient developed an infection at the graft harvest site.

Similar to the findings of Russell and Leighton [13], the incidence of complications in the bone graft donor site was 2.33% (1/43) in our study. Only one patient suffered lingering pain in the iliac bone-graft donor site for more than six months, but it resolved within the following 2 months. The mean VAS score for all patients was 0.21 at the last follow-up. Of note, the lower rate of complications was closely related to our method of bone harvest. The area where we harvested the bone segments was approximately 3.0 cm behind the ASIS and just anterior to the iliac tuberosity. If such a harvest is taken in this area, the risk of nerve or artery injury and fractures at the bone-graft donor site is reduced [36]. Structural bicortical ICBG (retaining the inner table) not only maintains the shape of the pelvis but also does not jeopardize the stability and strength of the crest [38]. In addition, completing haemostasis and suture reconstruction of the tissue plane are also essential for reducing the incidence of complications.

There are some limitations to this study. First, we only evaluated the postoperative clinical characteristics of DTPF patients and did not perform preoperative evaluation, which is a major limitation of our study. Second, small sample size and single-centre study may cause data bias to a certain extent. In addition, we did not include cases treated with other bone materials, bone graft donor sites, or fixation methods as control groups. In future studies, we will recruit more patients with DTPFs and set different control groups to further evaluate the clinical efficacy of structural bicortical autologous ICBG combined with TBTM.

## 5. Conclusion

The use of structural bicortical autologous ICBG combined with TBTM appears to be a promising option for treating DTPFs because it can provide better mechanical support and osteogenic potential, a good reduction and functional recovery, and relatively fewer complications.

## Figures and Tables

**Table 1:** Preoperative general data of patients.

Variables	*N* = 43 (mean ± SD/*n* (%))
Age (years (range))	46.28 ± 11.82 (21.00–69.00)
Gender (male)	30 (69.77)
BMI (kg/m^2^ (range))	27.20 ± 3.14 (21.27–34.12)
Affected side (left)	21 (48.84)
Smoking (yes)	6 (13.95)
Alcoholism (yes)	3 (6.98)
Mechanism of injury	
High energy	26 (60.47)
Low energy	17 (39.53)
Living area	
Rural	19 (44.19)
Urban	24 (55.81)
Hypertension (yes)	9 (20.93)
Diabetes (yes)	4 (9.30)
Follow-up time (months (range))	40.19 ± 7.58 (30.00–54.00)
Schatzker classification	
Type II	28 (65.12)
Type III	8 (18.60)
Type IV	7 (16.38)

Data are presented as the mean ± SD (range) or *n* (%); BMI: body mass index.

**Table 2:** Changes of intra-articular step-off at preoperation, postoperation, and final follow-up.

Variables	Preoperation	Immediate postoperation	Final follow-up
Intra-articular step-off (mm (range))	8.19 ± 4.05 (2.61–19.40)	1.30 ± 0.64^#^ (0.00–1.90)	1.48 ± 0.93^∗^ (0.00–4.97)

^#^The changes of intra-articular step-off between preoperation and immediate postoperation, *P* < 0.001; ∗the changes of intra-articular step-off between immediate postoperation and final follow-up, *P* = 0.108. Changes were assessed using paired *t*-test. *P* < 0.05 signified statistically significant difference.

**Table 3:** Clinical outcomes of bone graft donor site and bone graft site.

Variables	Bone-graft donor site (mean ± SD/*n* (%))	Bone-graft site (mean ± SD/*n* (%))
Incision length (cm (range))	4.08 ± 0.38 (3.50–5.00)	7.12 ± 0.60 (6.50–8.50)
Operative time (minutes (range))	8.95 ± 2.18 (6.00–15.00)	77.33 ± 22.50 (40.00–120.00)
Intraoperative blood loss (ml (range))	11.14 ± 2.80 (8.00–20.00)	126.74 ± 75.08 (50.00–300.00)
Union (yes)	43 (100.00)	43 (100.00)
Union time (months (range))	1.60 ± 0.62 (1.00–4.00)	2.45 ± 0.96 (2.00–9.00)
Postoperative malreduction rate	—	0 (0.00)
Secondary reduction loss rate	—	2 (4.65)
Complications	1 (2.33)	2 (4.65)
VAS score (final follow-up (range))	0.21 ± 0.56 (0.00–3.00)	0.09 ± 0.29 (0.00–2.00)
HSS score (final follow-up (range))	—	98.19 ± 2.98 (77.00–100.00)
SF-36 score (range)	95.65 ± 4.59 (75.00–100.00)	

VAS: Visual Analog Scale; HSS: Hospital for Special Surgery; SF-36: 36-Item Short-Form Health Survey.

## Data Availability

The data used to support the findings of this study are available from the corresponding author upon request.
